# Prevalence and molecular characterization of *Strongyloides stercoralis*, *Giardia duodenalis*, *Cryptosporidium* spp., and *Blastocystis* spp. isolates in school children in Cubal, Western Angola

**DOI:** 10.1186/s13071-018-2640-z

**Published:** 2018-01-29

**Authors:** Elena Dacal, José M. Saugar, Aida de Lucio, Marta Hernández-de-Mingo, Elena Robinson, Pamela C. Köster, María L. Aznar-Ruiz-de-Alegría, Mateu Espasa, Arlette Ninda, Javier Gandasegui, Elena Sulleiro, Milagros Moreno, Fernando Salvador, Israel Molina, Esperanza Rodríguez, David Carmena

**Affiliations:** 1 Parasitology Reference and Research Laboratory, National Centre for Microbiology, Health Institute Carlos III, Ctra. Majadahonda-Pozuelo Km 2, 28220 Majadahonda, Madrid Spain; 2 Hospital Nossa Senhora da Paz, Cubal, Benguela, Angola; 3 Microbiology Department, Vall d’Hebron University Hospital, PROSICS Barcelona, Passeig de la Vall d’Hebron 119-129, 08035 Barcelona, Spain; 4 Biomedical Research Institute of Salamanca - Research Center for Tropical Diseases at the University of Salamanca (IBSAL-CIETUS), Faculty of Pharmacy, University of Salamanca, Paseo de San Vicente 58-182, 37007 Salamanca, Spain; 5 Infectious Diseases Department, Vall d’Hebron University Hospital, PROSICS Barcelona, Passeig de la Vall d’Hebron 119-129, 08035 Barcelona, Spain

**Keywords:** *Strongyloides stercoralis*, *Giardia duodenalis*, *Cryptosporidium*, *Blastocystis*, Helminth, Nematode, Protozoa, Enteric parasites, Human, PCR, Molecular detection, Molecular epidemiology, Genotyping, Angola

## Abstract

**Background:**

Human infections by the gastrointestinal helminth *Strongyloides stercoralis* and the enteric protozoans *Giardia duodenalis*, *Cryptosporidium* spp. and *Blastocystis* spp. are not formally included in the list of 20 neglected tropical diseases prioritised by the World Health Organization. Although largely underdiagnosed and considered of lower public health relevance, these infections have been increasingly demonstrated to cause significant morbidity and even mortality globally, particularly among children living in resource-poor settings.

**Methods:**

In this cross-sectional survey the prevalence, frequency and molecular diversity of *S. stercoralis*, *G. duodenalis*, *Cryptosporidium* spp. and *Blastocystis* spp. were investigated in a school children population in the province of Benguela (Angola). A total of 351 stool samples were collected during January to June 2015. The presence of *S. stercoralis* and *G. duodenalis* was confirmed by qPCR methods. *Giardia duodenalis* assemblages and sub-assemblages were determined by multilocus sequence-based genotyping of the glutamate dehydrogenase and β-giardin genes of the parasite. Detection and identification of *Cryptosporidium* and *Blastocystis* species and subtypes was carried out by amplification and sequencing of a partial fragment of the small-subunit ribosomal RNA gene of both protozoan. Analyses of risk factors potentially associated with the transmission of these pathogens were also conducted.

**Results:**

Prevalences of *S. stercoralis*, *G. duodenalis*, *Cryptosporidium* spp., and *Blastocystis* spp. were estimated at 21.4% (95% CI: 17.1–25.7%), 37.9% (95% CI: 32.8–43.0%), 2.9% (95% CI: 1.1–4.5%) and 25.6% (95% CI: 21.18–30.2%), respectively. Overall, 64.1% (225/351) of the children were infected by at least one of the pathogens investigated. Sequence analyses of the 28 *G. duodenalis* isolates that were successfully genotyped allowed the identification of sub-assemblages AI (14.3%), AII (14.3%), BIII (7.1%) and BIV (25.0%). Discordant typing results AII/AIII and BIII/BIV were identified in 7.1% and 14.3% of the isolates, respectively. A total of five additional isolates (17.9%) were identified as assemblage B. Three *Cryptosporidium* species including *C. hominis* (70%), *C. parvum* (20%) and *C. canis* (10%) were found circulating in the children population under study. A total of 75 *Blastocystis* isolates were assigned to the subtypes ST1 (30.7%), ST2 (30.7%), ST3 (36.0%), ST5 (1.3%) and ST7 (1.3%), respectively. Children younger than seven years of age had significantly higher risk of infections by protozoan enteropathogens (PRR: 1.35, *P* < 0.01), whereas being underweight seemed to have a protective effect against these infections (PRR: 0.74, *P* = 0.005).

**Conclusions:**

The burden of disease attributable to human strongyloidiasis, giardiosis, cryptosporidiosis and blastocystosis in Angola is considerably higher than initially estimated in previous surveys. Surveillance and control of these infections should be jointly tackled with formally considered neglected tropical diseases in order to maximize effort and available resources. Our data also demonstrate the added value of using molecular diagnostic methods in high transmission areas.

**Electronic supplementary material:**

The online version of this article (10.1186/s13071-018-2640-z) contains supplementary material, which is available to authorized users.

## Background

Gastrointestinal helminthic and protozoan infections (GHPI) are significant contributors to the global burden of disease, particularly in low- and middle-income countries in tropical regions [1, 2]. GHPI primarily affect children in disfavoured settings with limited or no access to safe drinking water, inadequate sanitation, and substandard housing where poor hygiene practices are common [3]. Because enteric nematodes, cestodes, trematodes and protozoans often occur sympatrically in endemic areas, polyparasitism has been suggested to have a synergistic effect in exacerbating detrimental health outcomes in infected individuals [4]. Thus, GHPI have been linked to malabsorption, malnutrition, stunting, chronic anemia, cognitive impairment and failure to thrive [5–8]. Despite their unquestionable socio-economic and public health impact [9], the epidemiology of GHPI is still poorly understood in many regions of the world and only partially addressed in the Global Burden of Disease (GBD) studies [10].

Soil-transmitted helminths (STHs) including *Ancylostoma duodenale*, *Necator americanus*, *Ascaris lumbricoides*, *Trichuris trichiura* and *Strongyloides stercoralis* are estimated to infect almost one-sixth of the global population [9], with up to 370 million people being infected only with *S. stercoralis* [11]. Out of the approximately 50 species that comprise the genus *Strongyloides*, only *S. stercoralis* and *S. fuelleborni* are infective to humans, although the latter is clinically less important and has a restricted geographical distribution [12, 13]. As other STHs, *S. stercoralis* is primary transmitted through contact with soil that is contaminated with the nematode. Humans acquire the infection when filariform larvae come in contact with the skin, penetrate it, and migrate through the body. After reaching the small intestine, larvae mature into adult worms and lay their parthenogenetic eggs. Still within the intestinal lumen, the eggs hatch into non-infective rhabditiform larvae, which are excreted with stool into the environment. The larvae molt twice and then develop into infective filariform larvae re-initiating the cycle. Strongyloidiasis is an exception among helminthic infections because the parasite can reproduce within a human host (autoinfection) resulting in long-term infections and causing hyper-infection in debilitated and immunocompromised patients that can be fatal [14]. On the other hand, enteric protozoans *Cryptosporidium* spp., *G. intestinalis* and *Blastocystis* spp., together with *Entamoeba histolytica*, are regarded as the most common and important causes of protozoan-diarrhoeal disease in humans globally. These parasites are transmitted via the faecal-oral route either indirectly through ingestion of contaminated water or food or directly by contact with infected persons or animals. Only in developing countries, about 200 million people are estimated to have symptomatic giardiasis [15], whereas cryptosporidiosis is a leading cause of diarrhoeal death in children younger than 5 years globally, only second after rotaviral enteritis [10]. Regarding *Blastocystis* spp. it is currently estimated that up to 1 billion humans across the world would be colonized/infected with these protozoan species [16]. Although the clinical significance of *Blastocystis* spp. is still the focus of intense debate, there is mounting in vitro and in vivo evidence linking the presence of these protozoan species with intestinal (e.g. nausea, anorexia, flatulence, acute or chronic diarrhoea, irritable bowel syndrome) and extra-intestinal (e.g. urticarial) disorders [17, 18]. Moreover, invasive and inflammatory potential of the parasites has also been reported [19].

Furthermore, *Cryptosporidium* encompasses at least 30 valid species, of which *C. hominis* and *C. parvum* are known to cause more than 90% of documented human infections [20]. The only *Giardia* species that is pathogenic to humans, *G. intestinalis*, is currently regarded as a multi-species complex divided into eight (A to H) distinct genetic variants (assemblages) with marked differences in host range and specificity. Of these, zoonotic assemblages A and B are commonly reported to infect humans [21]. *Blastocystis* has also shown to exhibit extensive genetic diversity, allowing the differentiation of at least 17 subtypes (ST), of which STs 1–9 infect humans [22]. STs 10–17 are predominantly found in non-human species but some of them have been occasionally documented as zoonotic agents [23].

The current epidemiological situation of GHPI in Angola (southern Africa) remains largely unknown. Only a limited number of mainly microscopy-based studies targeting paediatric [8, 24–26] and women [24] populations have been conducted to date in the country. Obtained data revealed the presence of *A. lumbricoides* (4–17%), hookworms (0.6–14.0%), *T. trichiura* (0.3–14.0%) and *S. stercoralis* (0.3–3.5%) in the surveyed populations. Similarly, *G. duodenalis* and *Cryptosporidium* spp. were also identified at prevalence rates of 13–22% and 30% of the investigated individuals, respectively, although typing and sub-typing analyses were not carried out. In an attempt to overcome this situation, we present here PCR-based prevalence data of *S. stercoralis*, *G. duodenalis*, *Cryptosporidium* spp. and *Blastocystis* spp. in school children in Central Angola. Molecular data on the diversity and frequency of the protozoan species/genotypes investigated are also shown.

## Methods

### Study area

**Fig. 1:**
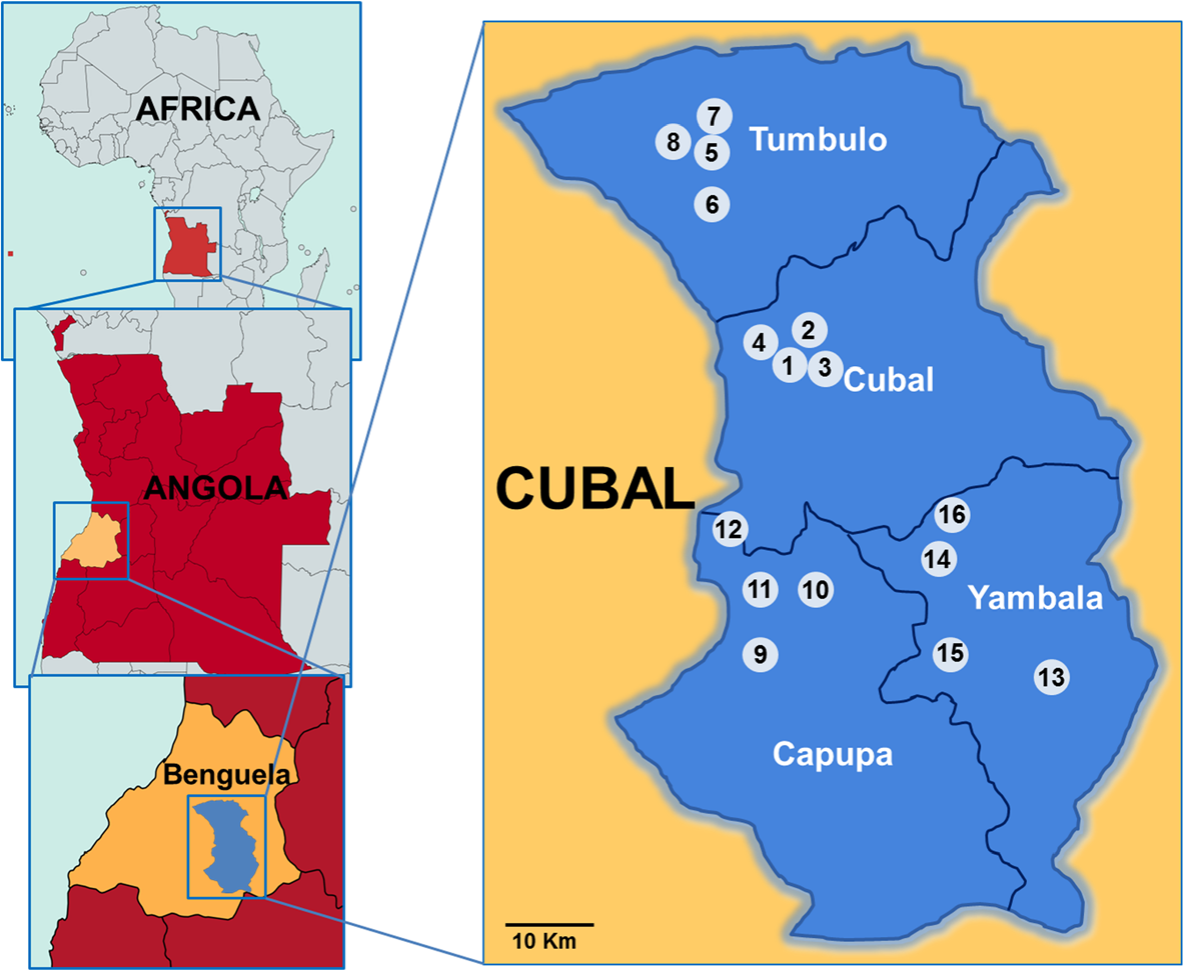
Map of Angola indicating the position of the province of Benguela. The exact location of the 16 schools visited in the four communes conforming the municipality of Cubal during the sampling campaign of the present study are also shown

Cubal is a municipality in the province of Benguela, in the highlands of the central plateau of Angola. The town is approximately 900 m above sea level and located 146 km from the city of Benguela. It extends over an area of 1452 km^2^ and has an estimated population of near 320,000 inhabitants, of whom 47% are children aged between 5 and 14. The municipality is administratively divided into four urban communes, namely Cubal, Tumbulo, Capupa and Yambala (Fig. 1). The dominant biome in Angola is the savannah. Like the rest of tropical Africa, Angola presents pronounced alternate wet-dry seasons of six-months. The interior highlands have a mild climate with a rainy season (November-April) followed by a dry season (May-October) [27, 28].

### Study design and stool sample collection

A cross-sectional study was conducted in primary schools in the four communes of Cubal from January to June 2015. The sample size was estimated at 378 children less than 15 year-old based on a 30% geohelminths prevalence previously reported in Angola [24, 29], a marginal error of 2.5%, and a non-response rate of 15%. Total population figures per commune were taken into account in the calculations. A two-step conglomerate sampling was carried out. First, the four most populated schools in each commune were identified and invited to participate in the present survey. Informative meetings were held with school representatives and parents to explain the goals of the project and the methodological aspects involved. Secondly, children were randomly selected to fill the numbers required in each sampled school. Children who received any antiparasitic drug treatment within the three previous months were excluded. Two consecutive stool specimens were obtained per child, individually collected in sterile polystyrene plastic flasks without any preservative, and suitably labelled. Socio-demographic (gender, age, commune of residence, school) and clinical (body mass index, haemoglobin levels) parameters were also compiled from each participant at the time of sample collection. Body mass index (BMI) values were adjusted for differences in gender and age using growth reference data for children and adolescents, 5–19 year- old [30]. Children whose BMI was less than the 5th percentile were considered underweight [31]. Following World Health Organization (WHO) guidelines, anaemia was defined as haemoglobin level of less than 11 g/dl [32].

Additionally, standardised data collection spreadsheets were used to identify risk factors potentially associated to the transmission of GHPI. Variables analysed included walking barefoot, household size, water-use practices (source of bath and drinking water, washing raw fruits and vegetables before eating), and contact with domestic animals. Samples were transported to the Microbiology Laboratory of the Nossa Senhora da Paz Hospital (Cubal) for parasite identification by coprological methods on the same day of collection. Aliquots of stool specimens were kept at -20 °C for further molecular analyses.

### DNA extraction and purification

Prior to DNA extraction, 1 g of each individual stool sample was re-suspended in 0.9% saline solution and concentrated using the Bioparaprep MINI® device (Leti Diagnostics, Barcelona, Spain), according to the protocol described elsewhere [33]. Total DNA was extracted from ~ 200 mg of concentrated faecal material using the QIAamp® DNA Stool Mini Kit (Qiagen, Hilden, Germany) following the manufacturer’s instructions. Purified DNA samples (200 μl) were stored at -20 °C and shipped to the Parasitology Service at the National Centre for Microbiology (Majadahonda, Spain) for downstream PCR-based diagnostic and genotyping analyses. In order to guarantee the absence of contamination of all the reagents used during the DNA extraction and purification procedure, a water extraction control was routinely included in each sample batch processed.

### Molecular detection of *Strongyloides stercoralis*

Genus-specific primers targeting the small subunit ribosomal RNA (*SSU* rRNA) gene of *Strongyloides* spp. [34] were used to detect the presence of the parasite in a qualitative real-time PCR (qPCR) assay using SybrGreen reagents (Invitrogen, San Diego CA, USA) as described elsewhere [33]. qPCR reactions were performed in a final volume of 25 μl containing 1× Quantimix EasyMaster Mix (Biotools B&M Laboratories, Madrid, Spain), 0.2 μM of each specific primer (Additional file 1: Table S1), 0.5 μl of 50× SybrGreen (Invitrogen, San Diego, CA, USA), and 10 μl of total DNA extracted from stool samples. Purified genomic DNA from *Strongyloides venezuelensis* L3 was used as positive control. All DNA isolates were assayed in duplicate. A third replicate including 10 ng of *S. venezuelensis* DNA was also included as internal inhibition control. Negative and no template controls were included in each run. The amplification program consisted of 15 min at 95 °C followed by 50 cycles of 10 s at 95 °C, 10 s at 60 °C and 30 s at 72 °C. DNA amplification and detection of fluorescence at the end of each amplification cycle was performed on a Corbett Rotor Gene™ 6000 real-time PCR system (Qiagen, Hilden, Germany). Data were analysed with Rotor Gene 6000 Series software version 1.7.

### Molecular detection and characterization of *Giardia duodenalis*

Initial detection of *G. duodenalis* DNA was achieved using a qPCR method targeting a 62 bp region of the *SSU* rRNA gene of the parasite [35]. Amplification reactions (25 μl) consisted of 3 μl of template DNA, 0.5 μM of primers Gd-80F and Gd-127R, 0.4 μM of probe (Additional file 1: Table S1), and 12.5 μl TaqMan® Gene Expression Master Mix (Applied Biosystems, CA, USA). Detection of parasitic DNA was performed on a Corbett Rotor Gene™ 6000 real-time PCR system (Qiagen) using an amplification protocol consisting on an initial hold step of 2 min at 55 °C and 15 min at 95 °C followed by 45 cycles of 15 s at 95 °C and 1 min at 60 °C. Water (no-template) and genomic DNA (positive) controls were included in each PCR run.

*Giardia duodenalis* isolates that tested positive by qPCR were subsequently assessed by sequence-based multi-locus genotyping of the genes encoding for the glutamate dehydrogenase (GDH) and ß-giardin (BG) proteins of the parasite. A semi-nested-PCR protocol proposed elsewhere [36] with minor modifications was used to amplify a ~ 432 bp fragment of the *gdh* gene. PCR reaction mixtures (25 μl) included 5 μl of template DNA and 0.5 μM of the primer pairs GDHeF/GDHiR in the primary reaction and GDHiF/GDHiR in the secondary reaction (Additional file 1: Table S1). Both amplification protocols consisted of an initial denaturation step at 95 °C for 3 min, followed by 35 cycles of 95 °C for 30 s, 55 °C for 30 s and 72 °C for 1 min, with a final extension of 72 °C for 7 min.

Similarly, a ~ 511 bp fragment of the *bg* gene of *G. duodenalis* was amplified using the nested-PCR protocol described by Lalle et al. [37]. PCR reaction mixtures (25 μl) consisted of 3 μl of template DNA and 0.4 μM of the primers sets G7_F/G759_R in the primary reaction and G99_F/G609_R in the secondary reaction (Additional file 1: Table S1). The primary PCR reaction was carried out with the following amplification conditions: 1 step of 95 °C for 7 min, followed by 35 cycles of 95 °C for 30 s, 65 °C for 30 s, and 72 °C for 1 min with a final extension of 72 °C for 7 min. The conditions for the secondary PCR were identical to the primary PCR except that the annealing temperature was 55 °C.

### Molecular detection and characterization of *Cryptosporidium* spp. isolates

The presence of *Cryptosporidium* spp. was assessed using a nested-PCR protocol to amplify a 587 bp fragment of the *SSU* rRNA gene of the parasite [38]. Amplification reactions (50 μl) included 3 μl of DNA sample and 0.3 μM of the primer pairs CR-P1/CR-P2 in the primary reaction and CR-P3/CPB-DIAGR in the secondary reaction (Additional file 1: Table S1). Both PCR reactions were carried out as follows: one step of 94 °C for 3 min, followed by 35 cycles of 94 °C for 40 s, 50 °C for 40 s and 72 °C for 1 min, concluding with a final extension of 72 °C for 10 min. Additionally, sub-typing of the isolates identified as *C. hominis* or *C. parvum* was attempted at the 60 kDa glycoprotein (*gp60*) locus following the nested-PCR protocol proposed by Feltus et al. [39].

### Molecular detection and characterization of *Blastocystis* spp. isolates

Identification of *Blastocystis* spp. was achieved by a direct PCR protocol targeting the *SSU* rRNA gene of the parasite [40]. This method uses the pan-*Blastocystis*, barcode primers RD5 and BhRDr (Additional file 1: Table S1) to amplify a PCR product of ~ 600 bp. Amplification reactions (25 μl) included 5 μl of template DNA and 0.5 μM of the primer set RD5/BhRDr. Amplification conditions consisted of one step of 95 °C for 3 min, followed by 30 cycles of 1 min each at 94, 59 and 72 °C, with an additional 2 min final extension at 72 °C.

All the direct, semi-nested, and nested PCR protocols described above were conducted on a 2720 thermal cycler (Applied Biosystems). Reaction mixes always included 2.5 units of MyTAQ™ DNA polymerase (Bioline GmbH, Luckenwalde, Germany), and 5× MyTAQ™ Reaction Buffer containing 5 mM dNTPs and 15 mM MgCl_2_. Laboratory-confirmed positive and negative DNA isolates for each parasitic species investigated were routinely used as controls and included in each round of PCR. PCR amplicons were visualized on 2% D5 agarose gels (Conda, Madrid, Spain) stained with Pronasafe nucleic acid staining solution (Conda). Positive-PCR products were directly sequenced in both directions using the internal primer set described above. DNA sequencing was conducted by capillary electrophoresis using the BigDye® Terminator chemistry (Applied Biosystems) on an on ABI PRISM 3130 automated DNA sequencer.

### Data analyses

The Chi-square test was used to compare *S. stercoralis*, *G. duodenalis*, *Cryptosporidium* spp., and *Blastocystis* spp. infection rates in the surveyed human population according to gender, age group, and place of residence. A probability (*P*) value < 0.05 was considered evidence of statistical significance. Prevalence risk ratios (PRR) and their 95% confidence intervals (CI) were calculated by univariate analyses to assess the association between potential risk factors considered in the individual data collection spreadsheets and infections with these helminth and protozoan pathogens. Data were analysed with the free software RStudio Version 1.0.44 (https://www.rstudio.com/) using the Epitools library.

### Sequence and phylogenetic analyses

Raw sequencing data in both forward and reverse directions were viewed using the Chromas Lite version 2.1 sequence analysis program (https://technelysium.com.au/wp/chromas/). The BLAST tool (http://blast.ncbi.nlm.nih.gov/Blast.cgi) was used to compare nucleotide sequences with sequences retrieved from the NCBI GenBank database. Generated DNA consensus sequences were aligned to appropriate reference sequences using the MEGA 6 software [41] to identify *Giardia* species and assemblages/sub-assemblages and *Cryptosporidium* species. *Blastocystis* sequences were submitted at the *Blastocystis* 18S database (http://pubmlst.org/blastocystis/) for sub-type confirmation and allele identification.

For the estimation of the phylogenetic inferences among the identified *Giardia*-positive samples, a phylogenetic tree was inferred using the Neighbor-Joining method in MEGA 6. Only representative, unambiguous (homozygous) sequences were used in the analyses. The evolutionary distances were computed using the Kimura 2-parameter method, and modelled with a gamma distribution. The reliability of the phylogenetic analyses at each branch node was estimated by the bootstrap method using 1000 replications. Representative sequences of the different *G. duodenalis* assemblages and sub-assemblages were retrieved from the GenBank database and included in the phylogenetic analysis for reference and comparative purposes. The sequences obtained in this study have been deposited in GenBank under accession numbers MF581531–MF581558 (*G. duodenalis*), MF581559–MF581564 (*Cryptosporidium* spp.) and MF581565– MF581576 (*Blastocystis* spp.).

## Results

Full sets of stool samples, epidemiological and clinical data, and signed informed consents were obtained from a total of 351 children. In 24.5% (86/351) of the participants only a single stool sample was collected due to field logistic problems. Additional file 2: Table S2 summarizes the main demographic features of the surveyed population. Briefly, 40.5% (142/351) of the children were recruited in Cubal, 23.9% (84/351) in Tumbulo, 14.0% (49/351) in Capupa, and 21.6% (76/351) in Yambala. The male/female ratio was 0.74. Overall, 28.8% (101/351) of the children fell in the age group 4–7 year-old, 51.3% (180/351) in the age group 8–11 year-old, whereas the remaining 19.9% (70/351) were aged between 12 and 15 years.

### Prevalence and risk factors for *Strongyloides stercoralis* infections

The prevalence of human strongyloidiasis, as determined by qPCR, was estimated at 21.4% [95% confidence interval (CI): 17.1–25.7%]. qPCR-positive results had cycle threshold (Ct) values ranging from 22.7 to 41.9 (median: 32.0). Males and females of all age groups were similarly affected, although those aged between 8 and 15 years harboured higher infection rates than younger children (Table 1). Attending to the frequency of detection of *Strongyloides* spp. by the commune of origin, the only statistically significant difference found was between children from Cubal and Capupa (*P* < 0.01). None of the variables considered in the study (gender, age group, walking barefoot, household size, bath place, contact with domestic animals, being moderately to severely underweight, and having anaemia) were associated with an increased risk of having strongyloidiasis (Table 2). Water-related practices including type of drinking water source or washing raw vegetables/fruits were not included in the analyses as these variables are not relevant for the transmission of the disease.

**Table 1:** Prevalence (%) and 95% confidence intervals (CIs) of the gastrointestinal helminths and protozoan species investigated in the present survey according to the gender, age group and commune of origin of the children population in Cubal, Angola, 2015. Variables showing statistically significant pairwise associations were identified with superscript (a to e) letters

		*n*	*Strongyloides* spp.	*Giardia duodenalis*	*Cryptosporidium* spp.	*Blastocystis* spp.
Global		351	21.4 (17.1–25.7)	37.9 (32.8–43.0)	2.9 (1.1–4.5)	25.6 (21.1–30.2)
Sex	Male	149	22.2 (15.5–28.8)	43.0 (35.0–50.9)	3.4 (−4.2–10.9)	28.2 (21.0–35.4)
	Female	202	20.8 (15.2–26.4)	34.2 (27.6–40.7)	2.5 (0.3–4.6)	23.8 (17.9–29.6)
Age group	4–7	101	15.8 (8.7–23.0)	54.5 (44.7–64.2)^b**^	4.0 (0.1–7.7)	20.8 (12.9–28.7)
	8–11	180	23.3 (17.2–29.5)	33.3 (26.4–40.2)^b**^	2.8 (0.4–5.2)	29.4 (22.8–36.1)
	12–15	70	24.3 (14.2–34.3)	25.7 (15.7–36.0)	1.4 (−1.4–4.2)	22.9 (13.0–32.7)
Commune	Cubal	142	28.9 (21.4–36.3)^a**^	37.3 (29.4–45.3)	3.5 (0.5–6.5)	16.9 (10.7–23.1)^c**, e*^
	Tumbulo	84	17.9 (9.7–26.1)	38.1 (27.7–48.5)	1.2 (−1.1–3.5)	39.3 (28.8–49.7)^c**, d*^
	Capupa	49	10.2 (1.7–18.7)^a**^	32.7 (19.5–45.8)	2.0 (−1.9–5.9)	20.4 (9.1–31.7)^d*^
	Yambala	76	18.4 (9.7–27.1)	42.1 (31.0–53.2)	4.0 (−0.4–8.3)	30.3 (19.9–40.6)^e*^

*P < 0.05; **P < 0.01

**Table 2:** Analysis of the variables identified as risk factors potentially involved in the transmission of *Strongyloides* spp. in Cubal, Angola, 2015. Prevalence risk ratios (PRR) and 95% confidence intervals (CIs) are indicated

Variable (no. of missing observations)	Category	Cases^a^	Non-cases^b^	PRR	95% CI	*P*
Gender (0)	Male	33	116	1.07	0.71–1.60	0.759
	Female	42	160	Reference		
Age group (0)	≤ 7 years old	16	85	0.67	0.41–1.11	0.108
	> 7 years old	59	191	Reference		
Walking barefoot (0)	Exposed	52	174	1.25	0.81–1.94	0.313
	Unexposed	23	102	Reference		
Household size (9)	> 6 individuals	37	123	1.17	0.78–1.76	0.451
	≤ 6 individuals	36	146	Reference		
Bath place (1)	Other^c^	37	156	0.79	0.53–1.18	0.253
	Home	38	119	Reference		
Contact with domestic animals (10)	Exposed	63	222	1.14	0.66–1.99	0.633
	Unexposed	12	50	Reference		
Body mass index^d^(10)	≤ P5	31	97	1.25	0.83–1.90	0.276
	> P5	41	172	Reference		
Haemoglobin level^d^(14)	≤ 11 g/dl	19	84	0.81	0.51–1.30	0.385
	> 11 g/dl	53	181	Reference		

^a^Cases: samples that tested positive for *Strongyloides* spp. by PCR-based methods

^b^Non-cases: samples that tested negative for *Strongyloides* spp. by PCR-based methods

^c^Street, river

^d^See Methods section

### Prevalence and risk factors for enteric protozoan infections

PCR-based results identified *G. duodenalis* as the most prevalent (37.9%; 95% CI: 32.8–43.0%) enteric protozoan parasite identified in the paediatric population under study, followed by *Blastocystis* spp. (25.6%; 95% CI: 21.18–30.2%), and *Cryptosporidium* spp. (2.9%; 95% CI: 1.1–4.5%) (Table 1). Gender was not significantly associated to blastocystosis, cryptosporidiosis, or giardiosis, although the later was found more often in male children than in female children. Individuals in the age group of 4–7 years harboured the greatest bulk of *G. duodenalis* and *Cryptosporidium* spp. infections. Indeed, children in that particular age group were found significantly more infected by *G. duodenalis* than children aged between eight and 11 years (*P* < 0.01). *Blastocystis* spp. affected children of all ages. Giardiosis or cryptosporidiosis were not statistically more prevalent in any given commune investigated, although differences in occurrence rates of blastocystosis were observed between children from Cubal and Tumbulo (*P* < 0.01), Cubal and Yambala (*P* < 0.05), and Tumbulo and Capupa (*P* < 0.05) (Table 1). Overall, children younger than seven years of age (PRR: 1.35, *P* < 0.01) were identified as the sub-population more exposed to infections by protozoan enteropathogens (Table 3). Interestingly, being underweight was found to be a protective factor against these infections (PRR: 0.74, *P* = 0.005). None of the remaining variables considered in the analysis (gender, household size, water-use practices, contact with domestic animals, and having anaemia) were linked to a higher risk of infection (Table 3).

**Table 3:** Analysis of the variables identified as risk factors potentially involved in the transmission of *Giardia duodenalis*, *Cryptosporidium* spp. and *Blastocystis* spp. in Cubal, Angola, 2015. Combined results for all protozoan parasites are shown. Prevalence risk ratios (PRR) and 95% confidence intervals (CIs) are indicated

Variable (no. of missing observations)	Category	Cases^a^	Non-cases^b^	PRR	95% CI	*P*
Gender (0)	Male	87	62	1.18	0.97–1.43	0.099
	Female	100	102	Reference		
Age group (0)	≤ 7 years old	66	35	1.35	1.12–1.63	0.004
	> 7 years old	121	129	Reference		
Household size (9)	> 6 individuals	78	82	0.86	0.70–1.06	0.147
	≤ 6 individuals	103	79	Reference		
Bath place (1)	Other^c^	111	82	1.20	0.98–1.47	0.069
	Home	75	82	Reference		
Contact with domestic animals (10)	Exposed	149	130	0.94	0.74–1.20	0.619
	Unexposed	35	27	Reference		
Drinking water source (0)	Other^d^	152	134	0.99	0.77–1.27	0.918
	Tap water	35	30	Reference		
Eating raw vegetables/fruits (0)	Yes	174	144	1.39	0.90–2.14	0.093
	No	13	20	Reference		
Raw vegetables/fruits washing (33)	No	37	31	1.00	0.78–1.28	0.998
	Yes	136	114	Reference		
Body mass index^e^ (10)	≤ P5	56	72	0.74	0.59–0.93	0.005
	> P5	126	87	Reference		
Haemoglobin level^e^ (14)	≤ 11 g/dl	60	43	1.14	0.92–1.40	0.237
	> 11 g/dl	120	114	Reference		

^a^Cases: samples that tested positive for *G. duodenalis*, *Cryptosporidium* spp. or *Blastocystis* spp. by PCR-based methods

^b^Non-cases: samples that tested negative for *G. duodenalis*, *Cryptosporidium* spp. or *Blastocystis* spp. by PCR-based methods

^c^Street, river

^d^Rainwater, surface water and groundwater

^e^See Methods section

### Single and multiple gastrointestinal helminthic and protozoan infections

The proportions of single or multiple infections involving gastrointestinal helminth or protozoan parasites in the children population are shown in Additional file 3: Table S3. Briefly, 64.1% (225/351) of the participants were infected by at least one of the pathogens investigated. *G. duodenalis* (30.7%; 69/225) followed by *Strongyloides* spp. (16.9%; 38/225) and *Blastocystis* spp. (16.9%; 38/225) were the parasite species most frequently found causing single infections. Similarly, co-infections by *G. duodenalis* + *Blastocystis* spp. (15.6%; 35/225) and *Strongyloides* spp. + *G. duodenalis* (9.3%; 21/225) were the dual combinations more commonly identified. Additionally, a total of six individuals (3%) harboured triple poliparasitisms by *Strongyloides* spp. + *G. duodenalis* + *Blastocystis* spp., and a single individual (0.4%) was found simultaneously infected by the four pathogen species.

### Molecular characterization of *G. duodenalis* isolates

A total of 133 DNA isolates tested positive for *G. duodenalis* by qPCR, providing Ct values within the range of 19.0–42.2 (median: 33.2). Of these, 17.3% (23/133) and 9.0% (12/133) were successfully amplified at the *gdh* and *bg* loci, respectively. A total of 28 isolates were genotyped and/or sub-genotyped by any of the two markers. Multi-locus genotyping data were available for 25.0% (7/28) of the isolates characterised. The low amplification rates obtained for the *gdh* and *bg* markers were found to be highly dependent of qPCR Ct values. Only seven *gdh* and four *bg* PCR amplicons were obtained from *G. duodenalis* isolates with qPCR Ct values > 30, which represented 72.9% (97/133) of the total.

Table 4 shows the diversity, frequency and main features of the *G. duodenalis* sequences generated at the *gdh* and *bg* loci in this survey. Sequence analyses revealed the presence of assemblages A (35.7%, 10/28) and B (64.3%, 18/28). No canine (C, D), feline (F), or ruminant (E) assemblages were detected. Out of the 10 assemblage A sequences, 14.3% (4/28) were assigned to the sub-assemblage AI and 14.3% (4/28) to the sub-assemblage AII. Ambiguous AII/AIII results were obtained for two isolates (7.1%; 2/28) at both the *gdh*/*bg* markers. Little genetic diversity was observed among AI-AIII isolates both at the *gdh* and *bg* loci, with most sequences (particularly within AII) being identical to their corresponding reference sequences. Single-nucleotide polymorphisms (SNPs) were detected at low rates in a limited number of sequences, some of them associated to amino acid substitutions and representing novel genotypes. Similarly, sequence analyses of the 18 isolates ascribed to the assemblage B allowed the identification of sub-assemblages BIII (7.1%; 2/28) and BIV (25.0%; 7/28). Discordant BIII/BIV typing results were determined in 14.3% (4/28) of the isolates at the *gdh* marker, whereas the remaining isolates (17.9%; 5/28) were allocated into the assemblage B at the *bg* (but not the *gdh*) locus. No inter-assemblage mixed infections were detected. Regardless of the genetic marker considered, virtually all B, BIII and BIV sequences differed among them by 4–8 SNPs, including a number of heterozygous positions in the form of double peaks in the electropherograms. Some of the generated sequences corresponded to genotypes not previously deposited in public repository databases.

**Table 4:** Diversity, frequency, and molecular features of *Giardia duodenalis* sequences at the *gdh* and *bg* loci obtained in the children population under study in Cubal, Angola, 2015. GenBank accession numbers are provided. Novel genotypes are underlined. Point mutations inducing amino acid substitutions are highlighted as superscript letters indicating the amino acid change

Locus	Assemblage	Sub-assemblage	Isolates	Reference sequence	Stretch	Single nucleotide polymorphisms	GenBank ID
*gdh*	A	AI	1	L40509	75–484	None	MF581531
			1	L40509	75–484	T126Y	MF581532
			1	L40509	75–484	T132A, C220A^a^	MF581533
			1	L40509	75–484	G191A^b^, T278C^c^	MF581534
		AII	5	L40510	78–482	None	MF581535
			1	L40510	78–482	T214Y	MF581536
		BIII	1	AF069059	40–460	C87T, T147C, G150A, C330T	MF581537
			1	AF069059	54–423	C99T, T147C, G150A, T276C, C309T, C336T, A412C^d^	MF581538
		BIV	2	L40508	64–442	None	MF581539
			1	L40508	77–482	G156R, T183Y, C273Y, T387Y, G408R, A438R	MF581540
			1	L40508	77–496	T183Y, G186R, C255T, C273T, T312Y, T387C, C423Y, A438R	MF581541
			1	L40508	78–482	T183C, T366C, T387C, A438G	MF581542
			1	L40508	77–496	T183C, T387C, C423T, A438G	MF581543
			1	L40508	78–482	T183Y, T366Y, T387C, C432Y	MF581544
		BIII/BIV	1	L40508	77–482	A122Y, T135C, T183Y, G186R, C255Y, C273Y, T366Y, T387Y, G408R, C411Y, A438R, A441R	MF581545
			1	L40508	76–496	T135Y, T183Y, G186R, C216Y, T222Y, C255Y, C273Y, T324Y, C345Y, T366Y, T387C, A438R, T462Y, T492Y	MF581546
			1	L40508	76–491	T135Y, T183Y, G186R, C255Y, C273Y, T312Y, C345Y, T366Y, C372Y, T387Y, C423Y, A438R	MF581547
			1	L40508	78–482	T183C, G186R, C255Y, C264Y, C273Y, T312Y, C345Y, T366Y, C372Y, G378R, T387Y, A438R	MF581548
*bg*	A	AIII	2	AY072724	102–590	None	MF581549
		B	1	AY072727	97–587	C114G, C117G, T240C, C309T	MF581550
			1	AY072727	102–593	C152T, C279T, C326T	MF581551
			1	AY072727	131–550	C165T, C168T	MF581552
			1	AY072727	103–590	A183G, A228G, C309T	MF581553
			1	AY072727	102–593	A183R, C309Y, C450Y	MF581554
			1	AY072727	103–589	A212G, C279T, A346W, A412R, A550R	MF581555
			1	AY072727	104–588	C279T	MF581556
			1	AY072727	102–601	C279T, A343G	MF581557
			1	AY072727	103–594	C309Y	MF581558
			1	AY072727	98–590	Poor quality sequence	–

M: A/C; R: A/G; W: A/T; Y: C/T

^a^p.P74T

^b^p.G64D

^c^p.L93P

^d^p.T138P

**Fig. 2:**
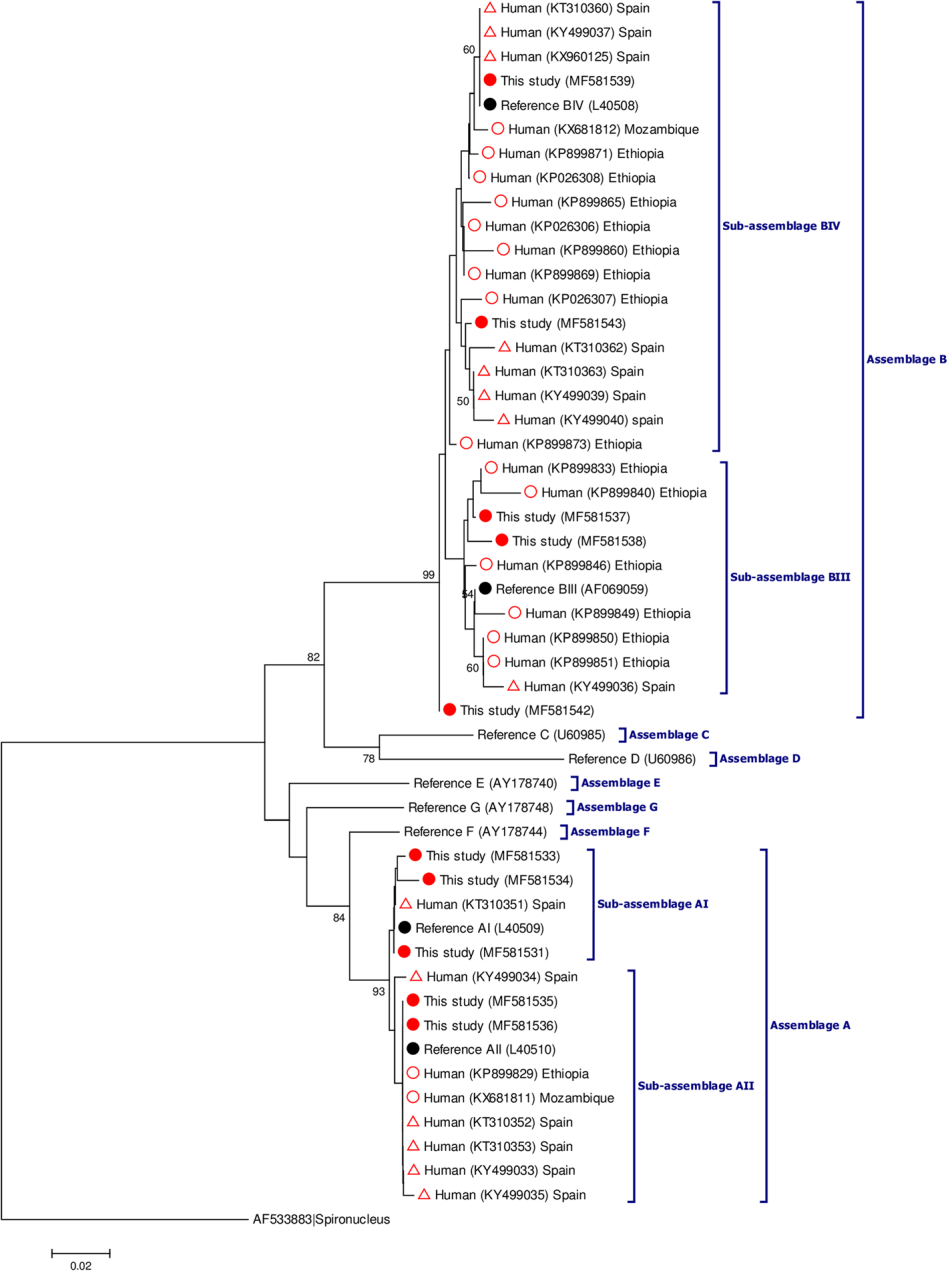
Phylogenetic tree depicting evolutionary relationships among *Giardia duodenalis* sequences at the *gdh* locus. The analysis was inferred using the Neighbor-Joining method of the nucleotide sequence covering a 353-bp region (positions 90–442 of GenBank accession L40508) of the gene. Bootstrap values lower than 50% were not displayed. Red filled circles represent sequences generated in the present study. Red empty circles and red empty triangles indicate sequences reported from other African countries and Spain, respectively, used for comparison purposes. Black filled circles represent reference sequences retrieved from the GenBank database. *Spironucleus vortens* was used as the outgroup

Figure 2 shows the phylogenetic relationships among the unambiguous (homozygous) sequences at the *gdh* locus obtained in the present study and reference sequences from NCBI. Sequences of human origin from developed (Spain) and developing (Ethiopia, Mozambique) countries generated hitherto in our laboratory were also included for comparison [42–46]. Sequences belonging to the assemblages A and B grouped together in well-defined clusters, even at the sub-assemblage (AI/AII or BIII/BIV) level. The exception was a BIV sequence (GenBank: MF581542) that did not comfortably fit within the BIII or BIV clades. Differences in branch length were noticeable in both BIII and BIV sequences, reflecting their comparatively elevated rate of nucleotide substitutions per site. Interestingly, most Spanish BIV sequences tended to form a sub-clade separated from BIV sequences from Angola, Ethiopia and Mozambique, an indication of potential variation in genotype prevalence between different geographical areas.

### Molecular characterization of *Cryptosporidium* spp. isolates

Analyses of *SSU* rDNA sequences demonstrated that human cryptosporidiosis in Cubal is primarily caused by *C. hominis* (70.0%; 7/10), with *C. parvum* (20.0%; 2/10) and *C. canis* (10.0%; 1/10) being much less frequently detected (Table 5). Among the seven sequences assigned to *C. hominis*, five showed 100% identity with the reference sequence AF108865, with the remaining two sequences corresponding to novel genotypes of the parasite with a variable number of SNPs including substitutions and the deletion of a single nucleotide. Alignment analyses of the two *Cryptosporidium* isolates identified as *C. parvum* allowed the identification of 366–489 bp fragments equivalent to positions 538–913 and 537–1025, respectively, of the reference sequence AF112571. Both sequences also represented genetic variants of *C. parvum* not previously described. Unfortunately, attempts to amplify the *C. hominis* and *C. parvum* isolates at the *gp60* locus failed repeatedly, so the sub-genotypes of the parasite involved in the children infections remain unknown. Finally, a single isolate was unambiguously assigned to the *Cryptosporidium* canine-specific species *C. canis*. Sequence analyses revealed that this isolate was identical to a stretch of sequence of 492 bp comprising positions 526–1017 of the reference sequence AF112576.

**Table 5:** Diversity, frequency, and molecular features of *Cryptosporidium* spp. sequences at the *SSU* rRNA locus obtained in the children population under study in Cubal, Angola, 2015. GenBank accession numbers are provided. Novel genotypes are underlined

Species	No. of isolates	Reference sequence	Stretch	Single nucleotide polymorphisms	GenBank ID
*C. hominis*	5	AF108865	574–994	None	MF581559
	1		534–1023	T623C, T692C, T695C, A946T	MF581560
	1		541–956	697delT^a^, T849A	MF581561
*C. parvum*	1	AF112571	538–913	A646G, T649G, A687T, 688_691delATTA^a^	MF581562
	1		537–1025	A646G, T649G, 686_689delTAAT^a^, T693A, C762T	MF581563
*C. canis*	1	AF112576	526–1017	None	MF581564

^a^del: nucleotide deletion(s)

**Fig. 3:**
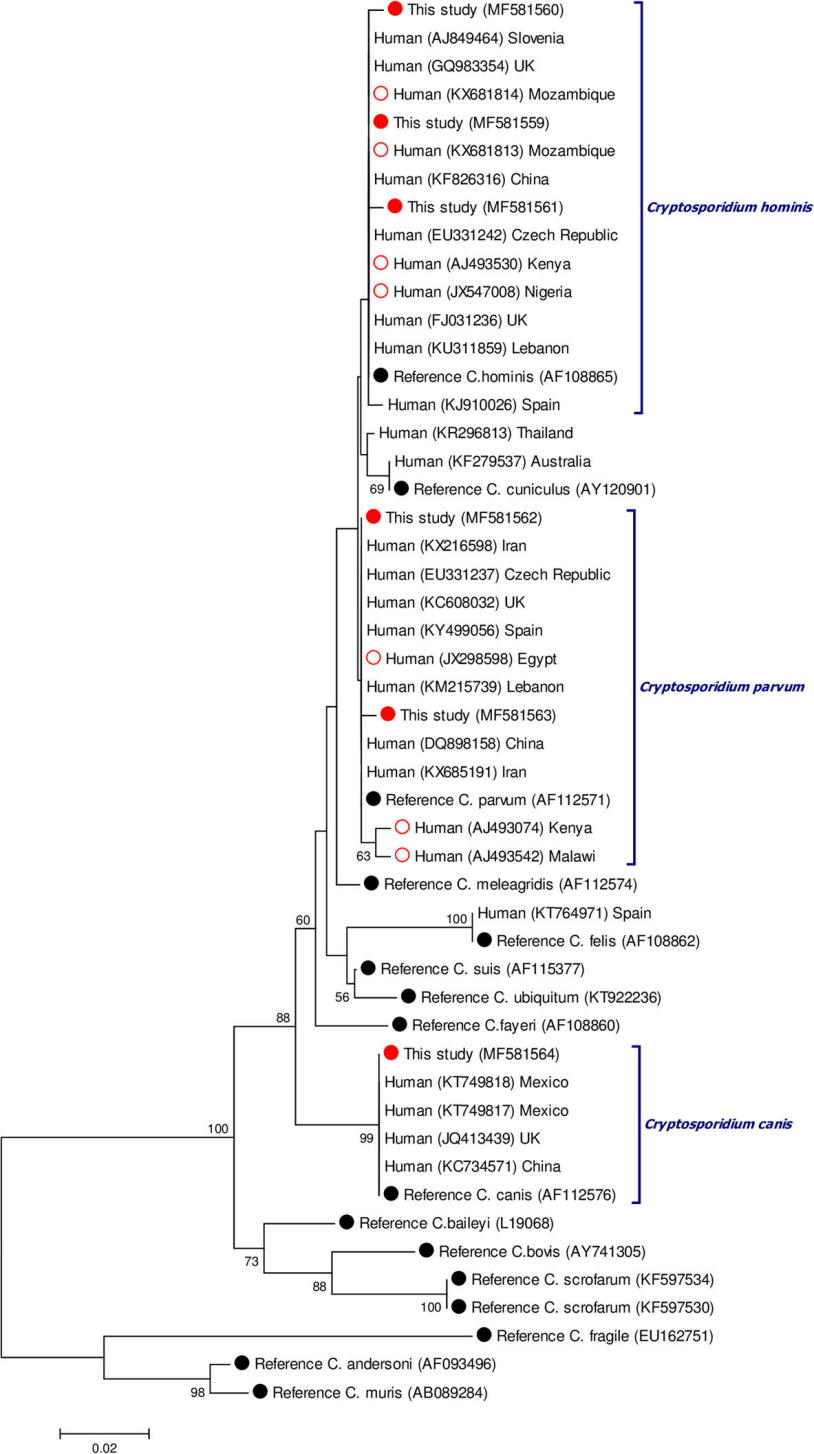
Phylogenetic tree depicting evolutionary relationships among *Cryptosporidium* spp. sequences at the *SSU* rRNA locus. The analysis was inferred using the Neighbor-Joining method of the nucleotide sequence covering a 416-bp region (positions 541–956 of GenBank accession AF108865) of the *C. hominis* gene. Bootstrap values lower than 50% were not displayed. Red filled circles represent sequences generated in the present study. Red empty circles indicate sequences reported from other African countries used for comparison purposes. Black filled circles represent reference sequences retrieved from the GenBank database

The phylogenetic tree depicted in Fig. 3 illustrates the inferred evolutionary history of the *SSU* rRNA gene sequences generated in the present study and those gathered from the NCBI database for reference and comparison purposes. As expected, sequences assigned to a specific *Cryptosporidium* species clustered together in well-defined clades demonstrating the robustness of our analyses.

### Molecular characterization of *Blastocystis* spp. isolates

**Fig. 4:**
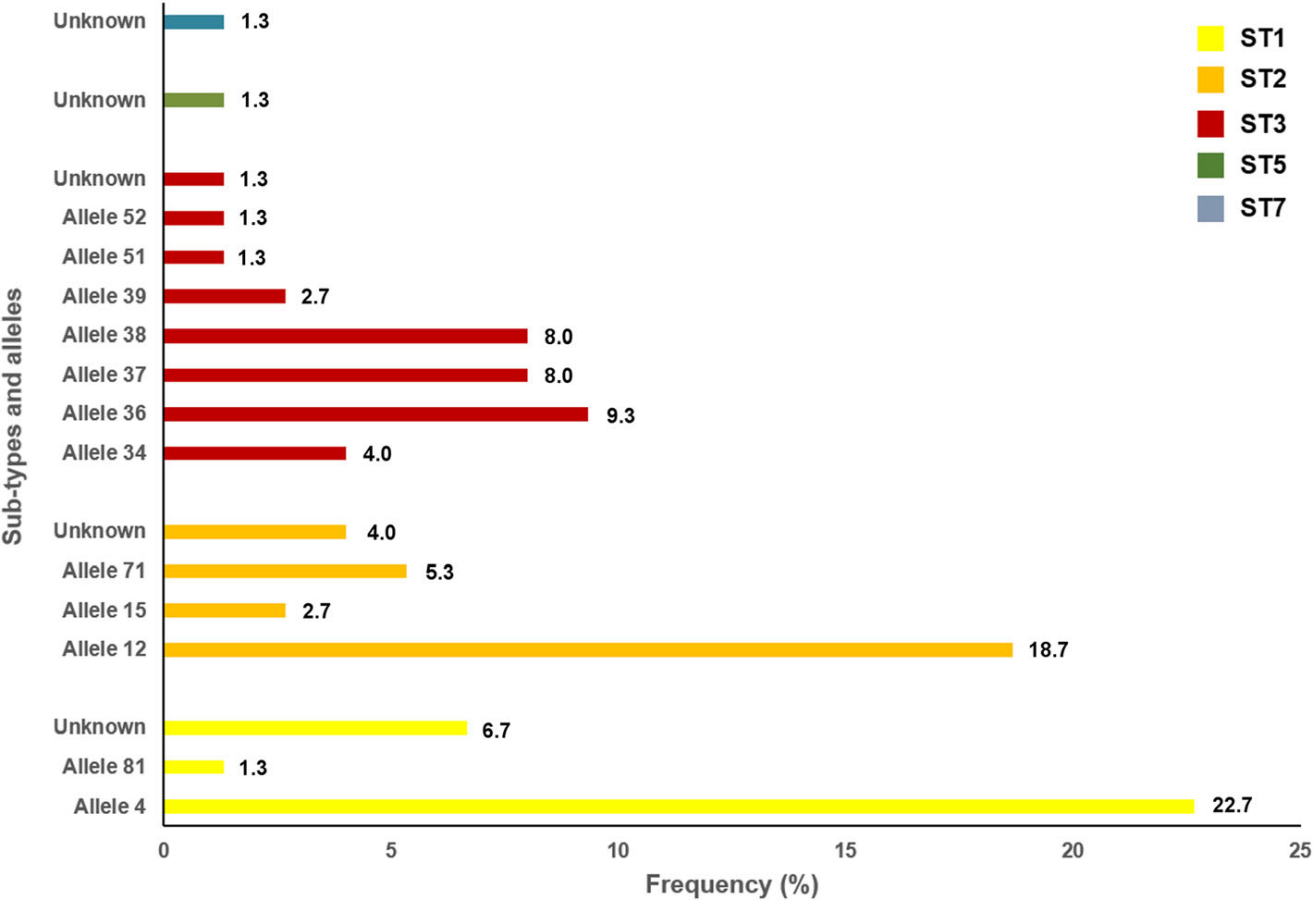
Diversity and frequency of *Blastocystis* subtypes and 18S alleles identified in the children population surveyed in Cubal, Angola, 2015

Out of the 90 isolates that tested positive for *Blastocystis* spp. by PCR, 83.3% (75/90) were successfully subtyped by sequence analyses at the *SSU* rDNA (barcode region) gene. BLAST searches allowed identification of five *Blastocystis* subtypes including ST1 (30.7%; 23/75), ST2 (30.7%; 23/75) and ST3 (36.0%; 27/75). Two additional isolates were recognized as ST5 and ST7 (1.3% each), respectively (Fig. 4). Neither mixed infection involving different STs of the parasite nor infections caused by animal-specific ST10-ST17 were recorded. Allele calling using the *Blastocystis SSU* database allowed the identification of alleles 4 and 81 within ST1, alleles 12, 15 and 71 within ST2, and alleles 34, 36, 37, 38, 39, 51 and 52 within ST3. Alleles 4 (22.7%; 17/75), 12 (18.7%; 14/75) and 36 (9.3%; 7/75) were found as the most represented in the human population under study. A number of isolates (five in ST1, three in ST2, and one in each ST3, ST5 and ST7) could not be analysed at the allele level due to inaccurate or incomplete sequencing data.

## Discussion

Neglected tropical diseases (NTDs) have been undeniably demonstrated as significant contributors to the global burden of disease, particularly in endemic areas from developing countries [47]. Since 2010 NTDs have received increasing attention and research funding from national and international agencies and institutions including WHO [48]. In contrast, other debilitating rather than fatal parasitic diseases have been often deemed less important in comparison to NTDs associated to higher morbidity/mortality rates. Epidemiological data from recent surveys, including the GBD 2010 initiative, have started to challenge this notion [49]. Thus, a number of helminthic and protozoan infections have emerged as pathogens with a much greater public health impact than initially anticipated. This is the case of *S. stercoralis*, a nematode not even strictly considered an STH [50], that have been proven to cause significant morbidity [11] and preventable mortality [14, 51] in numerous resource-poor tropical and subtropical countries [12]. Consequently, an expert recommendation has been made for the inclusion of *S. stercoralis* in prevalence studies targeting STH globally [52]. Similarly, the disease burden of cryptosporidiosis among young children has been estimated at almost 48 million disability-adjusted life years (DALYs), a figure comparable to that for tuberculosis (49 million) and more than half of the global burden of malaria (83 million) and HIV/AIDS (82 million) [49]. Although not formally an NTD, cryptosporidiosis (together with giardiosis) was included in the Neglected Diseases Initiative launched by WHO in 2004 [53]. In line with this global trend, molecular epidemiological data presented here clearly indicate that *Strongyloides* spp., *G. duodenalis*, *Cryptosporidium* spp., and (to a still uncertain extent), *Blastocystis* spp. have a substantial public health impact in Angola.

The overall prevalence of human strongyloidiasis obtained in our study (21.4%) was comparable to that (20.7%) previously documented in Ethiopia using a very similar diagnostic approach [54], but almost doubled the infection rate (12.8%) recently identified in the same Angolan children population by conventional and Baermann methods [26]. Of note, *Strongyloides* spp. has been undetected or reported at low (0.3–4%) infection rates in a number of epidemiological surveys conducted in Angola [8], Cameroon [55], Ethiopia [56, 57] and Kenya [58]. Taken together, these results highlight the convenience of using specific techniques (e.g. Baermann) and/or highly sensitive methods (e.g. PCR) for the accurate detection of the parasite in endemic areas. Interestingly, a PCR-based prevalence of 48% have been recently found in Mozambican general population [59], indicating that the prevalence of *Strongyloides* spp. increases with age, probably as a consequence of autoinfection and long-term (chronic) infection events. Our results seem to corroborate this age-related pattern, as most strongyloidiasis cases were found in children older than seven years.

Reported infection rates of giardiosis (0.1–62%) and cryptosporidiosis (0.1–72%) vary widely among African countries depending on the diagnostic method(s) of choice, the targeted sub-population (primarily children aged 0–16 years), and the immune status of the surveyed individuals [60]. The PCR-based prevalence (38%) for *G. duodenalis* found in the present study was substantially higher than those previously reported in other Angolan paediatric populations, including children presenting with diarrhoea in the Province of Bengo (21.6%) [25] and school children in the Province of Huíla (20.1%) [8]. These findings very likely reflect the superior diagnostic sensitivity of PCR over non-molecular methods. In contrast, a 30% *Cryptosporidium* infection rate was found in the later province using a commercial immunochromatographic rapid test, a 10-fold increase compared with the 2.9% prevalence for cryptosporidiosis identified in the present study by PCR. Variations in the epidemiology and transmission of the parasite or differences in the diagnostic specificity of the detection methods used may account for these discrepancies [61]. Although the epidemiology of blastocystosis in Africa is still poorly understood, a number of PCR-based diagnostic surveys seem to suggest that *Blastocystis* spp. infections are widespread. Prevalence rates of 61–100% have been reported in Nigeria [62], Senegal [63] and Tanzania [64]. Based on the same methodology, a much lower (but still considerable) infection rate of 25.6% was found in the children population under study, this being the first attempt to accurately estimate the prevalence of human blastocystosis in Angola.

A number of epidemiological, clinical, environmental, and behavioural factors have been linked to a higher risk of infections by GHPI. These include walking barefoot [56, 65, 66], having a low socio-economic status [67, 68], living in rural areas [68, 69], having contact with livestock [69–71], drinking untreated water [69, 72], eating unwashed/raw fruit [73], belonging to a given age group [25, 74], having diarrhoea [73], having a poor nutritional status [74, 75], and having anaemia [8, 73], among others. A number of studies have demonstrated that wearing shoes considerably reduce the risk of infection by STHs [56, 65, 66]. In our study none of the variables potentially involved in the transmission of *Strongyloides* spp. showed a significant association with the presence of the parasite, although the infection was more prevalent in people walking barefoot, reporting contact with domestic animals, or being part of households with six or more individuals. Similar results have been previously described in this very same population in a previous parasitological survey based on conventional techniques for the detection of the parasite [26]. Clearly, more rigorous epidemiological studies are needed to improve our understanding of the actual risk factors involved in the transmission of human strongyloidiasis. However, significantly different PCR-based prevalence rates of *Strongyloides* spp. were found between children in the communes of Cubal and Capupa, a finding not previously observed when the detection of the parasite was performed by conventional parasitological techniques [26]. These discrepancies may be attributed to differences in population densities leading to poorer hygienic habits (e.g. defecating in open spaces) or insufficient sanitation facilities (e.g. latrines, sewage treatment) in the most populated communes.

Children aged 4–7 years were found more vulnerable to *G. duodenalis* and *Cryptosporidium* spp. infections, with the former pathogen reaching statistical significance. Similar findings have been recurrently documented in other studies worldwide, being associated to the poor personal hygiene habits and immature cellular immunity of individuals in that particular age group. Interestingly, when all three protozoan parasites were collectively considered, infections were significantly less frequent in underweight children. This is in contrast with previous findings highlighting the important role of *G. duodenalis*, *Cryptosporidium* spp., and, to some extent, also *Blastocystis* spp. infections, as leading causes of poor absorption of nutrients, inadequate dietary intake, stunting and its associated cognitive and immunologic sequelae [76], features commonly seen in children from disfavoured settings in developing countries [5–8]. At present we do not have a clear explanation for this unexpected outcome. In this regard, iron deficiency linked with malnutrition has been associated with reduced odds of *G. duodenalis* chronic infection [77] and other infectious diseases including malaria [78, 79] in highly endemic areas. However, in the present study anaemia was not significantly associated with protection against enteric protozoan parasites. Clearly, more research should be conducted to elucidate this controversial point.

A high proportion (64%) of the children population studied was found infected by at least one enteric pathogen species. This percentage is very similar to that (67%) reported in north-western Angola [25] but considerably higher than that (44%) reported in the southern part of the country [8]. Overall, PCR-based prevalence data presented here clearly reveal that gastrointestinal helminthic and protozoan infections and co-infections are widespread in Cubal, Benguela. Based on their improved diagnostic performance and shorter turnaround time, our data also support the added benefit of adopting, when possible, molecular methods in high transmission areas, as previously demonstrated by other research groups [59].

Perhaps the major contribution of this study is the detailed account of the genetic diversity of *G. duodenalis*, *Cryptosporidium* spp. and *Blastocystis* spp. isolates from human origin in Angola, a task that had not been conducted to date. Sequence analyses of *G. duodenalis* isolates at both the *gdh* and *bg* loci revealed exciting data. Assemblage B (64%) had a higher prevalence than assemblage A (36%), proportions well in line with those documented in other African countries [21, 43, 44] and world regions [21, 80]. Confirming the results generated by our research group and others, a high level of genotypic diversity (evidenced by the presence of overlapping nucleotide peaks at specific positions in the corresponding sequencing profiles) was observed within assemblage B and, although to a much lesser extent, also within assemblage A. Consequently, virtually all sequences assigned to sub-assemblages AI, BIII and BIV represented distinct genotypic variants of *G. duodenalis*. In this regard, we have previously reported a much restricted degree of genotypic diversity in a large clinical cohort of patients with giardiosis in Spain, a developed country with a lower endemicity of the infection. In that survey most of the generated BIII and BIV sequences grouped together in a limited number of *G. duodenalis* genotypes [42]. We, therefore, envisage an epidemiological scenario for giardiosis in Cubal characterised by high infection pressure and transmission intensity of the parasite. Two independent mechanisms have been proposed to explain the large proportion of heterogeneous sequencing profiles commonly observed in assemblage B sequences: (i) the occurrence of true mixed infections, in which PCR amplification bias lead to the generation of sequences belonging to different assemblages/sub-assemblages of the parasite; or (ii) the occurrence of genetic recombination (e.g. intragenic recombination, exchange of alleles, nuclear fusion within cysts) leading to allelic sequence heterozygosity [80–82]. Because no intra-assemblage mixed infections were detected in the present study, we favoured recombination as the driving force behind the genetic variation observed in the assemblage B sequences from Angolan human isolates. Remarkably, four assemblage A sequences were confirmed as sub-assemblage AI at the *gdh* locus. In Africa, this particular genetic variant has only been identified in a limited number of human isolates from Algeria [83] and Egypt [84]. Within assemblage A, sub-assemblage AI is known to be responsible for 25% of human infections globally, this percentage being 62%–86% in livestock, 73% in domestic dogs and 44% in wildlife species [80]. Overall, it seems reasonable to think that some (if not all) of the AI infections found in this survey may have a zoonotic origin. Unfortunately, a lack of molecular data from animal sources precluded us from confirming this hypothesis.

*Cryptosporidium hominis* was confirmed as the most prevalent (70%) *Cryptosporidium* species causing human infections in Angola, followed by *C. parvum* (20%). These frequencies were in line with those previously reported by most epidemiological surveys in other African countries [60]. Of interest was the identification of novel genetic variants within both *C. hominis* and *C. parvum* at the *SSU* rRNA locus, a fact that may improve comparative sequence analyses that help understand the population dynamics of *Cryptosporidium* in the country. Available genotyping data seem to indicate that human cryptosporidiosis in Africa is primarily of anthroponotic origin. This assumption is based on two lines of evidence: (i) the typically anthroponotic *C. hominis* is the dominant *Cryptosporidium* species reported in African human populations; and (ii) most human infections with *C. parvum* are caused by the mainly anthroponotically transmitted IIc subtype of the parasite [60]. Our molecular results seem to support this notion, although the relatively low number of isolates analysed and the failure to characterize them at the *gp60* locus made advisable the confirmation of these findings in future studies. In contrast, solid evidence of zoonotic transmission was provided by the identification of the canine-specific *C. canis* infecting a child. Reflecting the opportunistic nature of these infections, *C. canis* has been sporadically documented in immunocompromised patients and young children in Ethiopia [71], Kenya [85] and Nigeria [86].

Notably, this survey provides novel data on the molecular epidemiology of *Blastocystis* spp. in Angola. The large set of genotyping results analysed here confirmed the presence at similar proportions of ST1, ST2 and ST3 in the children population investigated, with ST5 and ST7 causing marginal infections only. Very similar subtype frequencies have been documented in hospital outpatients in Tanzania [64]. In contrast, ST1 was the most prevalent *Blastocystis* subtype circulating among Nigerian school children [62] and Libyan outpatients [87], and ST3 in both asymptomatic and clinical populations in Egypt [88, 89], Senegal [63] and Tunisia [90]. Noticeably, ST4 was not detected in the investigated paediatric population. This *Blastocystis* subtype is commonly seen in European countries such as Denmark [91], France [92], Sweden [93] and the UK [94], but rare or absent in African countries [63, 64, 90, 94], evidencing marked differences in subtype distribution by geographical region and (very likely) between groups of individuals. Although not yet conclusively proven, there is mounting evidence suggesting that *Blastocystis* pathogenicity could be subtype-related. For instance, ST1 has been linked to the aetiology of irritable bowel syndrome [95], whereas ST3 [96] and ST4 [91, 97] have been predominantly found in patients with gastrointestinal disorders, mainly diarrhoea. Definitively, more research should be conducted in this field to ascertain the potential correlation between ST and virulence. Regarding transmission, recent phylogenetic analyses have demonstrated that most human ST3 infections fell into specific clades and are the consequence of human-to-human transmission [98]. On the other hand, ST5 is common in pigs [99] and ST7 in ground-dwelling birds [100], but considered rare in humans [94]. These findings suggest that the ST5 and ST7 infections detected here may be the result of zoonotic transmission events, although the extent of this possibility should be further confirmed in studies targeting both domestic and wildlife animal species in Angola.

A number of technical limitations may have hampered the accuracy of the diagnostic and genotyping results presented here. For instance, the high Ct values observed in a large proportion of the samples that tested positive to *Strongyloides* spp. and *G. duodenalis* by qPCR were somehow unexpected in a theoretically endemic area such as Cubal. This fact could be associated to sub-optimal extraction and purification of genomic DNA from stool samples, or inadequate conservation of DNA isolates prior to PCR testing. If true, reported infection rates may be an underestimation of the actual prevalence of GHPI in this geographical area. This could also explain, at least partially, the relatively low percentage of *G. duodenalis* isolates genotyped at the sub-assemblage level, or the failure to determine *C. hominis*/*C. parvum* subtypes at the *gp60* marker.

## Conclusions

This is the most thorough study assessing the current epidemiological situation of human strongyloidiasis, giardiosis, cryptosporidiosis, and blastocystosis conducted in Angola to date. Our molecular data clearly demonstrate that the burden of illness associated to these neglected parasitic diseases is higher than initially estimated in the country. Taking into consideration that these GHPI share several common features regarding transmission, surveillance, diagnosis, and treatment with other STHs formally considered as NTDs (case of *S. stercoralis*) and diarrhoea-causing pathogens (case of *G. duodenalis*, *Cryptosporidium* spp. and *Blastocystis* spp.) it seems wise and advisable to adopt a more holistic and integrated approach to tackle the control and eradication of these diseases jointly, particularly in deprived areas in developing countries. This strategy will undoubtedly contribute to maximize effort and resources devoted to the task.

## Additional files

Additional file 1: Table S1. Oligonucleotides used for the molecular identification and/or characterization of *Strongyloides stercoralis*, *Giardia duodenalis*, *Cryptosporidium* spp. and *Blastocystis* spp. in this study. (DOCX 14 kb)

Additional file 2: Table S2. Main demographic features of the children population recruited in the present study (*n* = 351) in Cubal, Angola, 2015. (DOCX 14 kb)

Additional file 3: Table S3. Frequency of single and multiple infections by enteric helminthic and protozoan parasites in Cubal, Angola, 2015. (DOCX 14 kb)

## Abbreviations

BG: ß-giardin
CI: confidence interval
DALY: disability-adjusted life year
GBD: global burden of disease
GDH: glutamate dehydrogenase
GHPI: gastrointestinal helminthic and protozoan infections
GP60: 60-kDa glycoprotein
NTD: neglected tropical disease
PRR: prevalence risk ratio
qPCR: real-time polymerase chain reaction
*SSU* rRNA: small subunit ribosomal RNA
ST: subtype
STH: soil-transmitted helminth
WHO: World Health Organization
