# Supplementary material for: Prevalence and molecular characterization of Strongyloides stercoralis, Giardia duodenalis, Cryptosporidium spp., and Blastocystis spp. isolates in school children in Cubal, Western Angola
Source: Parasit Vectors. 2018 Jan 29;11:67. doi: 10.1186/s13071-018-2640-z (PMC5789528; doi:10.1186/s13071-018-2640-z)
Supplement: Supplementary file 1 — Oligonucleotides used for the molecular identification and/or characterization of Strongyloides stercoralis, Giardia duodenalis, Cryptosporidium spp. and Blastocystis spp. in this study. (DOCX 14 kb) [file 13071_2018_2640_MOESM1_ESM.docx]

**Additional file 1: Table S1.** Oligonucleotides used for the molecular identification and/or characterization of *Strongyloides stercoralis*, *Giardia duodenalis*, *Cryptosporidium* spp., and *Blastocystis* spp. in this study.

| Target organism | Locus | Oligonucleotide | Sequence (5´–3´) | Reference |
| --- | --- | --- | --- | --- |
| *Strongyloides stercoralis* | *SSU* rRNA | F | GAATTCCAAGTAAACGTAAGTCATTAGC | [34] |
|  |  | R | TGCCTCTGGATATTGCTCAGTTC | [34] |
| *Giardia duodenalis* | *SSU* rRNA | Probe | FAM–CCCGCGGCGGTCCCTGCTAG–BHQ1 | [35] |
|  |  | Gd-80F | GACGGCTCAGGACAACGGTT | [35] |
|  |  | Gd-127R | TTGCCAGCGGTGTCCG | [35] |
|  | *gdh* | GDHeF | TCAACGTYAAYCGYGGYTTCCGT | [36] |
|  |  | GDHiF | CAGTACACCTCYGCTCTCGG | [36] |
|  |  | GDHiR | GTTRTCCTTGCACATCTCC | [36] |
|  | *bg* | G7_F | AAGCCCGACGACCTCACCCGCAGTGC | [37] |
|  |  | G759_R | GAGGCCGCCCTGGATCTTCGAGACGAC | [37] |
|  |  | G99_F | GAACGAACGAGATCGAGGTCCG | [37] |
|  |  | G609_R | CTCGACGAGCTTCGTGTT | [37] |
| *Cryptosporidium* spp. | *SSU* rRNA | CR-P1 | CAGGGAGGTAGTGACAAGAA | [38] |
|  |  | CR-P2 | TCAGCCTTGCGACCATACTC | [38] |
|  |  | CR-P3 | ATTGGAGGGCAAGTCTGGTG | [38] |
|  |  | CPB-DIAGR | TAAGGTGCTGAAGGAGTAAGG | [38] |
|  | *gp60* | AL-3531 | ATAGTCTCCGCTGTATTC | [39] |
|  |  | Al-3535 | GGAAGGAACGATGTATCT | [39] |
|  |  | AL-3532 | TCCGCTGTATTCTCAGCC | [39] |
|  |  | AL-3534 | GCAGAGGAACCAGCATC | [39] |
| *Blastocystis* spp. | *SSU* rRNA | BhRDr | GAGCTTTTTAACTGCAACAACG | [40] |
|  |  | RD5 | ATCTGGTTGATCCTGCCAGT | [40] |
