# Supplementary material for: Prevalence and molecular characterization of Strongyloides stercoralis, Giardia duodenalis, Cryptosporidium spp., and Blastocystis spp. isolates in school children in Cubal, Western Angola
Source: Parasit Vectors. 2018 Jan 29;11:67. doi: 10.1186/s13071-018-2640-z (PMC5789528; doi:10.1186/s13071-018-2640-z)
Supplement: Supplementary file 2 — Main demographic features of the children population recruited in the present study (n = 351) in Cubal, Angola, 2015. (DOCX 14 kb) [file 13071_2018_2640_MOESM2_ESM.docx]

**Additional file 2: Table S2.** Main demographic features of the children population recruited in the present study (*n* = 351) in Cubal, Angola, 2015.

|  |  |  | **Gender** | | **Age group** | | | |
| --- | --- | --- | --- | --- | --- | --- | --- | --- |
| **Commune** | **School** | **Nº of children** | **Male** | **Female** | **4‒7** | **8‒11** | **12‒15** |  |
| Cubal | García Neto | 24 | 10 | 14 | 6 | 15 | 3 |  |
|  | Tomas Ferreira | 33 | 13 | 20 | 11 | 18 | 4 |  |
|  | Comandate Bula | 56 | 26 | 30 | 20 | 25 | 11 |  |
|  | Teresianas | 29 | 7 | 22 | 12 | 17 | 0 |  |
| Tumbulo | Tumbulo | 19 | 9 | 10 | 5 | 9 | 5 |  |
|  | Kaliamuma | 23 | 7 | 16 | 6 | 11 | 6 |  |
|  | Kambindji | 18 | 10 | 8 | 7 | 6 | 5 |  |
|  | Lulambo | 24 | 8 | 16 | 3 | 12 | 9 |  |
| Capupa | Hoji-Ha-Henda | 3 | 3 | 0 | 1 | 2 | 0 |  |
|  | Loneta Halu | 24 | 12 | 12 | 5 | 14 | 5 |  |
|  | Cassua | 13 | 5 | 8 | 4 | 6 | 3 |  |
|  | Caviva Sul | 9 | 2 | 7 | 0 | 6 | 3 |  |
| Yambala | Yambala | 19 | 10 | 9 | 8 | 9 | 2 |  |
|  | Kasalasitu | 19 | 8 | 11 | 9 | 6 | 4 |  |
|  | Kambondongolo | 24 | 11 | 13 | 4 | 16 | 4 |  |
|  | Atiopo | 14 | 8 | 6 | 0 | 8 | 6 |  |
| Total |  | 351 | 149 | 202 | 101 | 180 | 70 |  |
