# Supplementary material for: Prevalence and molecular characterization of Strongyloides stercoralis, Giardia duodenalis, Cryptosporidium spp., and Blastocystis spp. isolates in school children in Cubal, Western Angola
Source: Parasit Vectors. 2018 Jan 29;11:67. doi: 10.1186/s13071-018-2640-z (PMC5789528; doi:10.1186/s13071-018-2640-z)
Supplement: Supplementary file 3 — Frequency of single and multiple infections by enteric helminthic and protozoan parasites in Cubal, Angola, 2015. (DOCX 14 kb) [file 13071_2018_2640_MOESM3_ESM.docx]

**Additional file 3: Table S3.** Frequency of single and multiple infections by enteric helminthic and protozoan parasites in Cubal, Angola, 2015.

|  | **Cases (*n*)** | **Percentage (%)^a^** | **Percentage (%)^b^** |
| --- | --- | --- | --- |
| **Single infections** (*n* = 150) |  |  |  |
| *Strongyloides* spp. | 38 | 16.89 | 10.83 |
| *Giardia duodenalis* | 69 | 30.67 | 19.66 |
| *Cryptosporidium* spp. | 5 | 2.22 | 1.42 |
| *Blastocystis* spp. | 38 | 16.89 | 10.83 |
| **Double infections** (*n* = 68) |  |  |  |
| *Strongyloides* spp. + *Giardia duodenalis* | 21 | 9.33 | 5.98 |
| *Strongyloides* spp. + *Cryptosporidium* spp. | 1 | 0.44 | 0.28 |
| *Strongyloides* spp. + *Blastocystis* spp. | 8 | 3.56 | 2.28 |
| *Giardia duodenalis* + *Cryptosporidium* spp. | 1 | 0.44 | 0.28 |
| *Giardia duodenalis* + *Blastocystis* spp*.* | 35 | 15.56 | 9.97 |
| *Cryptosporidium* spp. *+ Blastocystis* spp. | 2 | 0.89 | 0.57 |
| **Triple infections** (*n* = 6) |  |  |  |
| *Strongyloides* spp. + *Giardia duodenalis + Blastocystis* spp. | 6 | 2.67 | 1.71 |
| **Quadruple infections** (*n* = 1) |  |  |  |
| *Strongyloides* spp. + *Giardia duodenalis + Cryptosporidium* spp. + *Blastocystis* spp. | 1 | 0.44 | 0.28 |

^a^Over the total individuals infected by at least one enteric pathogen (*n* = 225).

^b^Over the total individuals surveyed (*n* = 351).
